# Age- and sex-specific associations between sarcopenia severity and poor cognitive function among community-dwelling older adults in Japan: The IRIDE Cohort Study

**DOI:** 10.3389/fpubh.2023.1148404

**Published:** 2023-04-04

**Authors:** Takahisa Ohta, Hiroyuki Sasai, Yosuke Osuka, Narumi Kojima, Takumi Abe, Mari Yamashita, Shuichi P. Obuchi, Tatsuro Ishizaki, Yoshinori Fujiwara, Shuichi Awata, Kenji Toba, IRIDE Cohort Study Investigators

**Affiliations:** ^1^Integrated Research Initiative for Living Well With Dementia, Tokyo Metropolitan Institute for Geriatrics and Gerontology, Tokyo, Japan; ^2^Research Team for Promoting Independence and Mental Health, Tokyo Metropolitan Institute for Geriatrics and Gerontology, Tokyo, Japan; ^3^Department of Frailty Research, Center for Gerontology and Social Science, Research Institute, National Center for Geriatrics and Gerontology, Obu, Japan; ^4^Research Team for Social Participation and Community Health, Tokyo Metropolitan Institute for Geriatrics and Gerontology, Tokyo, Japan; ^5^Human Care Research Team, Tokyo Metropolitan Institute for Geriatrics and Gerontology, Tokyo, Japan; ^6^Tokyo Metropolitan Institute for Geriatrics and Gerontology, Tokyo, Japan

**Keywords:** sarcopenia, cognitive function, poor cognitive function, Asian Working Group for Sarcopenia 2019, sarcopenia severity

## Abstract

**Introduction:**

This study examined whether the association between sarcopenia severity and cognitive function differed according to sex and age in community-dwelling older adults in Japan.

**Methods:**

This is a cross-sectional study of older adults (age ≥ 65 years) consisting of five regional cohorts integrated as the Integrated Research Initiative for Living Well with Dementia (IRIDE) Cohort Study. Sarcopenia severity was determined based on the Asian Working Group for Sarcopenia 2019, which assessed grip strength, walking speed, and skeletal muscle mass index. Poor cognitive function was defined as a Mini-Mental State Examination score of ≤ 23. Odds ratios (ORs) and 95% confidence intervals (CIs) for poor cognitive function were calculated by sex and age group (65–74 and ≥75 years) using binomial logistic regression models, which were adjusted for age, educational attainment, history of non-communicable diseases, smoking and drinking habits, living alone, frequency of going outdoors, exercise habits, and depressive symptom.

**Results:**

Of the 8,180 participants, 6,426 (1,157 men aged 65–74 and 1,063 men aged 75 or older; 2,281 women aged 65–74 and 1,925 women aged 75 or older) were analyzed. The prevalence ratio of sarcopenia and severe sarcopenia were 309 (13.9%) and 92 (4.1%) among men and 559 (13.3%) and 166 (3.7%) among women, respectively. A total of 127 (5.8%) men and 161 (3.9%) women had a poor cognitive function. Setting non-sarcopenia as a reference, the adjusted ORs (95% CI) of poor cognitive function were 2.20 (1.54, 3.15) for sarcopenia and 3.56 (2.20, 5.71) for severe sarcopenia. A similar trend was observed in analyses stratified by sex and age, with linear associations (*P* for trend <0.05) in both categories. Furthermore, there was a significant interaction (*P* < 0.05) between sex and sarcopenia severity, indicating a stronger linear association of sarcopenia severity with poor cognitive function in women compared with men.

**Discussion and conclusion:**

Sarcopenia severity was linearly associated with poor cognitive function in adults aged ≥ 65 years, with a stronger association in women compared with men.

## Introduction

Cognitive decline leading to dementia or mild cognitive impairment (MCI) is characterized by decreased memory, language, and executive function abilities. The number of dementia patients worldwide is projected to reach 78 million by 2030 and 139 million by 2050 (1). In addition, the number of dementia patients in the super-aged society of Japan is estimated to reach 7.4 million in 2030 and 8.5 million in 2060 (2). Therefore, examining the factors that may lead to poor cognitive function is essential.

Risk factors of poor cognitive function include genetic, psychological, and environmental factors, such as physical inactivity (3). Physical inactivity increases the risk of cognitive decline resulting from cardiovascular disease (4), and promotes the accumulation of amyloid-β and tau proteins specific to Alzheimer's disease (5). In contrast, myokines, such as brain-derived neurotrophic factor (BDNF), are secreted by muscle cells with the contraction of skeletal muscles in regular exercise. These myokines cross the blood-brain barrier and promote further BDNF production in the brain. This, in turn, promotes improved neurogenesis, memory, and learning, suggesting that myokines may be effective in improving cognitive function (5, 6). Therefore, since physical activity is closely related to skeletal muscle performance (7), the quantity, quality, and function of skeletal muscle may substantially impact cognitive function.

From an epidemiological perspective, the association between sarcopenia and MCI has been reported in numerous observational studies and systematic reviews (8–10). Sarcopenia is defined as an age-related decline in skeletal muscle mass, muscle strength, and physical function, with decreased physical activity and poor nutrition as risk factors (11). As evidence suggests a close relationship between reduced muscle mass and strength and neuroinflammatory responses in the cerebrum, many individuals with sarcopenia may experience a cognitive decline (12, 13). However, the associations between sarcopenia and cognitive function may be controversial due to methodological issues such as insufficient sample sizes, and no consistent conclusions have been obtained (8, 9). In addition, the relationship between sex- and age-related sarcopenia and cognitive function in Japan's super-aged society, particularly with regard to cognitive decline in severely physically impaired sarcopenic individuals, has not been examined, and these findings may serve as a guideline for the realization of healthy longevity.

This study aimed to examine the association between sarcopenia and cognitive function, and its specificity by sex and age, in a large sample of community-dwelling older Japanese adults. These findings may provide a valid basis for detecting cognitive decline according to skeletal muscle characteristics in older populations.

## Methods

### Study design and participants

This study analyzed cross-sectional data from the Integrated Research Initiative for Living Well With Dementia Cohort Study (IRIDE-CS) conducted by the Tokyo Metropolitan Institute of Gerontology (14). The IRIDE-CS included five cohorts of community-dwelling older adults (≥65 years) from the Otassha Study (*n* = 3,426), Takashimadaira Study (*n* = 2,053), Septuagenarians, Octogenarians, Nonagenarians Investigation with Centenarians (SONIC) Study (*n* = 567), Hatoyama Study (*n* = 742), and Kusatsu Longitudinal Study on Aging (*n* = 1,392). Each cohort is an ongoing longitudinal study with its own recruitment methods (15–19). A total of 8,180 older adults were included in the IRIDE-CS.

All participants were informed about the aims and protocols of each cohort study as well as the IRIDE-CS, and written informed consent was obtained. This study was conducted in accordance with the Declaration of Helsinki and was approved by the Research Ethics Committee of the Tokyo Metropolitan Institute for Geriatrics and Gerontology (R21–28).

### Assessment of sarcopenia

Sarcopenia status was based on lean mass, muscle strength, and physical function, and was defined according to the criteria established by the 2019 Asian Working Group for Sarcopenia (AWGS) (11). Lean mass was assessed using direct segmental multi-frequency bioelectrical impedance analysis (InBody S10, Biospace, Seoul, Korea for Takashimadaira cohort, InBody 720 analyzer, InBody Co., Ltd., Seoul, Korea for the other cohorts). Low muscle mass was defined as a skeletal muscle mass index of <7.0 kg/m^2^ for men and <5.7 kg/m^2^ for women. Low muscle strength was defined as handgrip strength of 28 kg for men and < 18 kg for women. Handgrip strength was assessed with Takei 5401 Digital Dynamometer (Takei, Japan). Low physical function was defined as a 5-m gait speed of <1.0 m/s for both sexes. Sarcopenia was defined as low muscle mass, and either low muscle strength or low physical function. Severe sarcopenia was defined as low muscle mass and strength as well as low physical function.

### Definition of poor cognitive function

Cognitive function was assessed using the Mini-Mental State Examination, which assessed the following: time registration, place registration, immediate and delayed recall of three words, mathematical calculation, object naming, sentence recall, three levels of verbal commands, written commands, sentence writing, and pentagon drawing (20). The cutoff for MMSE was set at 24 points, at which substantial hippocampal atrophy may be detected (21).

### Assessment of covariates

Information on the history of non-communicable diseases (hypertension, diabetes, dyslipidemia, and stroke) was obtained using a questionnaire with yes/no answers in self-reported form and interview. Data on smoking status (never/past/current), drinking status (never/past/current), educational attainment (years), frequency of going outdoors (days per week) (<1, 1–2, 3–6, 7), exercise habits (days per week; <1, 1–4, >4), and depressive symptoms [Geriatric Depression Scale-15 (GDS-15)] were also obtained (22).

### Statistical analysis

Participants were divided by sex and age (65–74 and ≥ 75 years). Continuous variables, which performed normality tests, were expressed as means (standard deviations) or median [interquartile range], and categorical variables were expressed as numbers and percentages.

The association between sarcopenia severity and poor cognitive function was evaluated using binary logistic regression analyses. Multivariable odds ratios (ORs) and 95% confidence intervals (CIs) were calculated using non-sarcopenia as a reference after adjusting for age, educational attainment, smoking and drinking status, cohort categories, living alone, history of non-communicable diseases (hypertension, diabetes, dyslipidemia, and stroke), GDS-15 scores, frequency of going outdoors, and exercise habits. Sex- and age-stratified analyses were also performed. In addition, the linearity association was tested by entering sarcopenia severity as a continuous variable in a regression model. Finally, to test whether sex differences in the association between sarcopenia severity and cognitive function existed, an interaction term (sex^*^sarcopenia severity) was created and added to the multivariate model for analysis.

Data were analyzed using SPSS Statistics version 25.0 (IBM Corporation, Armonk, NY, USA), and statistical significance was set at *p* < 0.05.

## Results

**Figure 1:**
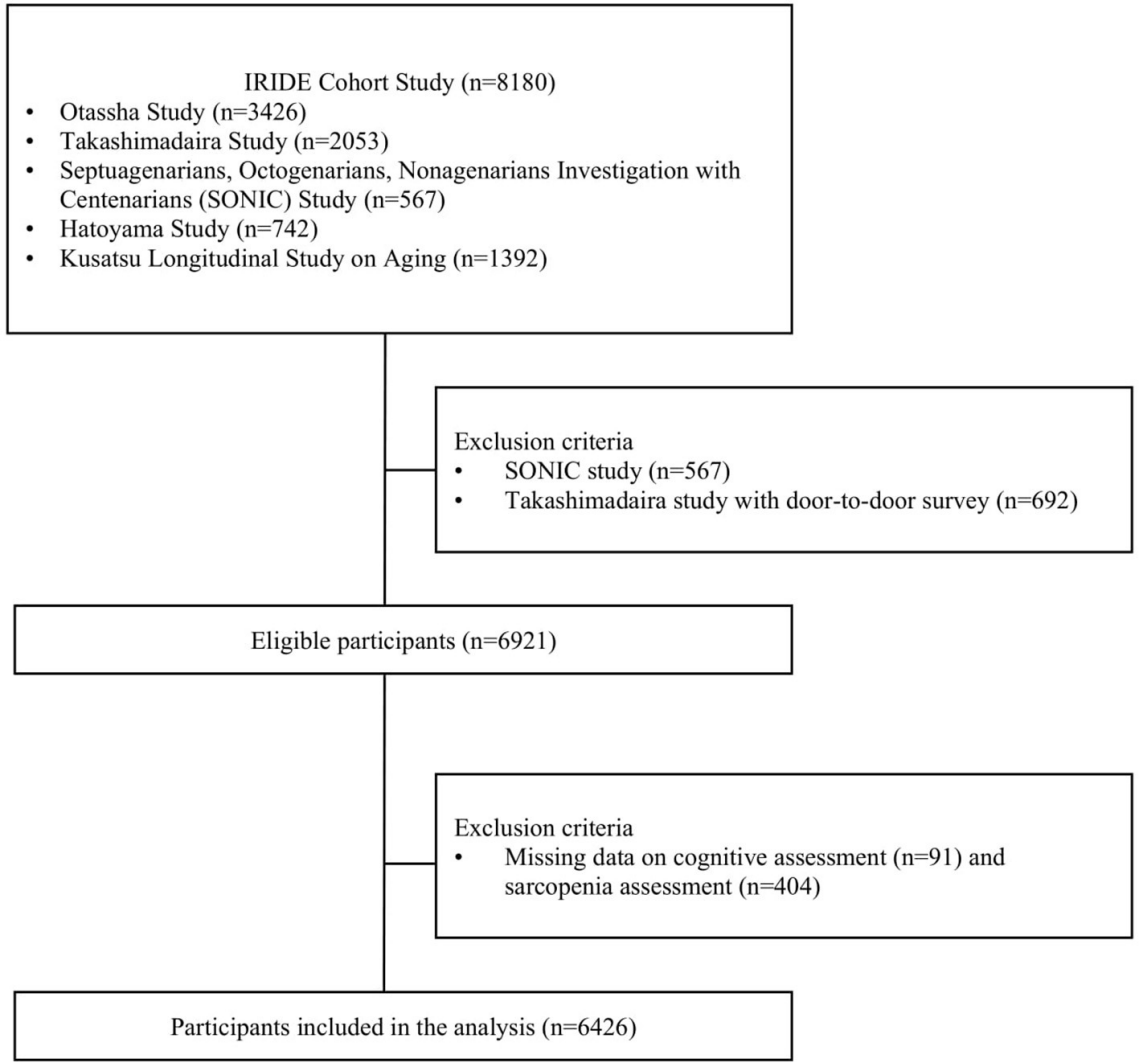
Flowchart of participant selection.

Some of the SONIC and Takashimadaira studies were excluded from the analysis because sarcopenia could not be determined (e.g., missing body composition and gait function). In addition, those with missing MMSE scores (*n* = 91) and sarcopenia status (*n* = 404) were excluded. In total, 6,426 participants were included in the analysis (Figure 1).

The characteristics of the cohort [mean age (min-max): 72.9 (65–95) years] are shown in Table 1. The prevalence of sarcopenia was 13.9% for men and 13.3% for women. The prevalence of severe sarcopenia was 4.2% for men and 3.9% for women. Poor cognitive function was observed in 270 participants (4.2%), with a higher prevalence in men (5.7%) compared with in women (3.4%). Educational attainment was higher in men than in women and decreased with sarcopenia severity in both sexes.

**Table 1:** Characteristics of the cohort according to the severity of sarcopenia.

		**Men (*****n*** = **2,220)**			**Women (*****n*** = **4,206)**		
		**Sarcopenia status**			**Sarcopenia status**		
	**Overall (*****n*** = **6,426)**	**Non-sarcopenia (*****n*** = **1,819, 81.9%)**	**Sarcopenia (*****n*** = **309, 13.9%)**	**Severe sarcopenia (*****n*** = **92, 4.2%)**	**Non-sarcopenia (*****n*** = **3,481, 82.8%)**	**Sarcopenia (*****n*** = **559, 13.3%)**	**Severe sarcopenia (*****n*** = **166, 3.9%)**
Age, years	74 [9]	73 [10]	79 [7]	83 [6]	73 [9]	78 [8]	80 [8]
65–74	3,438 (53.5)	1,078 (59.3)	70 (22.7)	9 (9.8)	2,081 (59.8)	180 (32.2)	20 (12.0)
≥75	2,988 (46.5)	741 (40.7)	239 (77.3)	83 (90.2)	1,400 (40.2)	379 (67.8)	146 (88.0)
MMSE, score	29 [3]	29 [2]	28 [4]	27 [4]	29 [2]	28 [2]	27 [4]
Poor cognitive function, yes	270 (4.2)	74 (4.1)	34 (11.0)	19 (20.7)	75 (2.2)	35 (6.3)	33 (19.9)
Past medical history							
Hypertension	2,849 (44.3)	880 (48.4)	145 (46.9)	49 (53.3)	1,439 (41.3)	249 (44.5)	87 (52.4)
Diabetes	811 (12.6)	322 (17.7)	62 (20.1)	20 (21.7)	328 (9.4)	57 (10.2)	22 (13.3)
Dyslipidemia	2,257 (35.1)	510 (28.0)	78 (25.2)	22 (23.9)	1,373 (39.4)	227 (40.6)	47 (28.3)
Stroke	377 (5.9)	154 (8.5)	23 (7.4)	16 (17.4)	137 (3.9)	32 (5.7)	15 (9.0)
Smoking							
Current	624 (9.7)	312 (17.2)	54 (17.5)	13 (14.1)	201 (5.8)	35 (6.3)	9 (5.4)
Past	1,623 (25.3)	968 (53.2)	162 (52.4)	52 (56.5)	370 (10.6)	52 (9.3)	19 (11.4)
Never	4,065 (63.3)	505 (27.8)	89 (28.8)	23 (25.0)	2,865 (82.3)	459 (82.1)	124 (74.7)
Alcohol consumption							
Current	2,844 (44.3)	1,190 (65.4)	173 (56.0)	34 (37.0)	1,265 (36.3)	143 (25.6)	39 (23.5)
Past	518 (8.1)	172 (9.5)	54 (17.5)	20 (21.7)	206 (5.9)	50 (8.9)	16 (9.6)
Never	2,881 (44.8)	393 (21.6)	66 (21.4)	29 (31.5)	1,955 (56.2)	341 (61.0)	97 (58.4)
Educational attainment, years	12 [4]	12 [4]	12 [7]	12 [7]	12 [3]	12 [3]	11 [3]
GDS-15, score	2 [3]	2 [3]	3 [5]	4 [4]	2 [3]	3 [5]	4 [4]
Depressive symptoms, *n*	1,244 (19.4)	313 (17.2)	96 (31.4)	30 (32.6)	568 (16.3)	180 (32.2)	57 (34.3)
Frequency of going outdoors (days per week)							
< 1	39 (0.6)	12 (0.7)	3 (1.0)	3 (3.3)	12 (0.3)	3 (0.5)	6 (3.6)
1–2	192 (3.0)	49 (2.7)	13 (4.2)	8 (8.7)	67 (1.9)	34 (6.1)	21 (12.7)
3–6	1,269 (19.7)	310 (17.0)	78 (25.2)	26 (28.3)	665 (19.1)	148 (26.5)	42 (25.3)
7	4,902 (76.3)	1,445 (79.4)	213 (68.9)	52 (56.5)	2,727 (78.3)	370 (66.2)	95 (57.2)
Exercise habits (days per week)							
< 1	888 (13.8)	246 (13.5)	41 (13.3)	13 (14.1)	471 (13.5)	89 (15.9)	28 (16.9)
1–4	5,068 (78.9)	1,380 (75.9)	237 (76.7)	65 (70.7)	2,842 (81.6)	427 (76.4)	117 (70.5)
>4	86 (1.3)	49 (2.7)	2 (0.6)	1 (1.1)	30 (0.9)	2 (0.4)	2 (1.2)

Data are means (standard deviations) or median [interquartile range], or numbers (percentages).

MMSE, Mini-mental State Examination; GDS, Geriatric Depression Scale-15.

Depressive symptoms were defined as a GDS ≥ 5.

There was a positive association between sarcopenia severity and poor cognitive function (*P* for trend < 0.001). The multivariable OR (95% CIs) for poor cognitive function was 2.19 (1.54, 3.13) for individuals with sarcopenia and 3.43 (2.14, 5.49) for those with severe sarcopenia (Table 2).

**Table 2:** Sex-stratified odds ratios (95% confidence intervals) of poor cognitive function according to sarcopenia status and gender.

	**Age-stratified**	**Number of cases (%)**		**Sarcopenia**	**Severe sarcopenia**	***P* for trend**	**Interaction between sex and sarcopenia severity**
All participants (*n* = 6,426)		270 (4.2)	Unadjusted	3.16 (2.37, 4.21)	9.91 (7.11, 13.81)	< 0.001	
			Age-adjusted	2.05 (1.52, 2.77)	4.50 (3.12, 6.51)	<0.001	
			Multivariate^†^	2.19 (1.54, 3.13)	3.43 (2.14, 5.49)	<0.001	
Men	Total cohort (*n* = 2,220)	127 (5.7)	Unadjusted	2.92 (1.91, 4.46)	6.14 (3.52, 9.74)	<0.001	
			Age-adjusted	1.93 (1.22, 3.06)	2.80 (1.48, 5.30)	<0.001	
			Multivariate^*^	2.00 (1.14, 3.51)	2.78 (1.24, 6.23)	0.003	
	65–74 years (*n* = 1,157)	35 (3.0)	Age-adjusted	5.12 (2.16, 12.12)	4.71 (0.55, 40.02)	0.002	
			Multivariate^*^	3.74 (1.03, 13.60)	18.27 (1.29, 259.69)	0.007	
	≥75 years (*n* = 1,083)	92 (8.7)	Age-adjusted	1.45 (0.87, 2.42)	2.70 (1.42, 5.13)	0.005	
			Multivariate^*^	1.73 (0.92, 3.24)	2.41 (1.02, 5.70)	0.021	[-20,-30]72pt0.001
Women	Total cohort (*n* = 4,206)	143 (3.4)	Unadjusted	3.37 (2.28, 4.96)	13.50 (8.88, 20.52)	<0.001	
			Age-adjusted	2.00 (1.31, 3.07)	5.36 (3.26, 8.79)	<0.001	[-15,-30]72pt0.001
			Multivariate^*^	2.36 (1.48, 3.76)	4.18 (2.31, 7.56)	<0.001	<0.001
	65–74 years (*n* = 2,281)	28 (1.2)	Age-adjusted	3.05 (1.22, 7.63)	13.97 (3.75, 51.98)	<0.001	
			Multivariate^*^	3.57 (1.29, 9.88)	15.68 (2.97, 82.93)	0.001	
	≥75 years (*n* = 1,925)	115 (6.0)	Age-adjusted	1.99 (1.27, 3.10)	5.15 (3.16, 8.41)	<0.001	
			Multivariate^*^	2.04 (1.21, 3.44)	3.57 (1.87, 6.80)	<0.001	

The odds ratios and 95% confidence intervals were calculated using non-sarcopenia as a reference in each model.

^†^Adjusted for sex, age, educational attainment, smoking status, drinking status, cohort categories, living alone, history of non-communicable diseases (hypertension, diabetes, dyslipidemia, and stroke), geriatric depressive symptom (GDS ≥ 5), frequency of going outdoors, and exercise habits.

^*^, † minus sex.

The sex- and age-stratified analyses showed that prevalence of poor cognitive function was higher in the older age group than in the younger age group for both sexes. Additionally, the prevalence of poor cognitive function was higher in men (57.2%) than that in women (34.0%) and higher in older category (86.5% for men and 59.7% for women). Multivariate analyses showed a positive association between sarcopenia severity and poor cognitive function in both sexes and age-category. Its association was stronger in women than men (*p* for interaction; <0.001) between sex and sarcopenia severity in both age groups.

## Discussion

This study investigated the association between sarcopenia severity and poor cognitive function according to sex and age among community-dwelling older adults in Japan. There was a linear relationship between sarcopenia severity and poor cognitive function across different sex and age categories and was most clearly reflected in the older age group. Additionally, this association was stronger in women compared with men. These findings indicate that the association between sarcopenia severity, or skeletal muscle health, and cognitive function is clearer for women than for men. Additionally, these results suggest that skeletal muscle mass and function and motor control may be closely associated with cognitive function and, through related behavior change, may serve as a method for preventing MCI and dementia.

Systematic reviews and meta-analyses have reported an association between sarcopenia and cognitive impairment, but the heterogeneity is high and inconclusive (8, 9). Our results showed that severe sarcopenia was associated with a higher risk of poor cognitive function than sarcopenia and non-sarcopenia in both sexes and age categories, consistent with a previous study demonstrating a strong association between poor physical function and cognitive impairment (23). Therefore, not only muscle mass, but also muscle strength and gait function may contribute to cognitive function. The role of skeletal muscle as an endocrine organ includes the secretion of myokines with its contraction in exercise. For instance, physical activity and exercise stimulate BDNF expression in the hippocampus, a brain region responsible for memory and learning (24). This association is widely recognized as muscle-brain crosstalk, and quantitative and qualitative (neurological) aspects of skeletal muscle are closely related to poor cognitive function (6, 25). Indeed, aerobic and resistance exercise interventions have been shown to be effective in improving cognitive function, regardless of cognitive status (26). Other possibilities include an increased fear of falling and injury due to reduced skeletal muscle function, which may result in a shift away from social participation and a decline in cognitive function (27). Alternatively, the progression of sarcopenia could lead to vascular cognitive dysfunction *via* exacerbation of metabolic diseases (28). Therefore, further longitudinal studies from multiple perspectives on the relationship between sarcopenia and cognitive function are needed.

The detailed mechanisms for the sex differences in the effects of sarcopenia severity on cognitive function are unclear, although they may be related to biological differences between men and women, such as differences in brain volume (29, 30), sex hormones (31), and body composition and physical performance (31). In particular, brain volume is related to brain reserve and is known to be one of the factors contributing to sex differences in cognitive decline (32). It has also been shown that elevated inflammatory markers (e.g., interleukin-6 and C-reactive protein) associated with reduced skeletal muscle mass are particularly noticeable in women (33). These may be one factor explaining the possible strong association between sarcopenia severity and poor cognitive decline in women than in men (29).

Moreover, the correlation between sarcopenia and poor cognitive function may be due to inflammation, oxidative stress, and abnormal hormone secretion (34). These conditions affect skeletal muscle and brain health and may contribute to brain atrophy in neurodegenerative diseases (35). In addition, inflammatory biomarkers, such as tumor necrosis factor-α, interleukin-6, and C-reactive protein, are associated with physical function and medial temporal lobe atrophy (36, 37). Therefore, future longitudinal studies should be conducted to ascertain whether inflammatory markers mediate the association between sarcopenia and poor cognitive function.

We clearly demonstrated linear association between sarcopenia severity and poor cognitive function according to sex and age categories. This can be attainable due to larger sample size than that of previous studies and systematic reviews. However, there are several noteworthy limitations. First, the study's cross-sectional and observational nature prevented the determination of causal relationships between sarcopenia and poor cognitive function. Second, although the model adjusted for potential confounding factors, unmeasured confounders may have affected the association, including dietary habits, and genetic factors. Third, the voluntary nature of participation in health checkups may have led to selection bias, where the individuals who underwent checkups tended to have higher health literacy levels, and those who did not might have had additional health conditions or lower health literacy levels. Finally, poor cognitive function was assessed using the MMSE, and a cutoff with high sensitivity and specificity for MCI was applied. However, the MMSE is not necessarily valid for diagnosing dementia or MCI. Therefore, some participants with poor cognitive function may have been misclassified. Future studies should incorporate methods higher sensitivity levels for monitoring cognitive outcomes during examinations.

## Conclusion

In conclusion, sarcopenia severity was linearly associated with poor cognitive function among community-dwelling older adults in Japan, and this association was stronger in women compared with men. Further studies should examine the longitudinal relationship between sarcopenia and poor cognitive function, including the onset of MCI and dementia.

## Data availability statement

The raw data supporting the conclusions of this article will be made available by the authors, without undue reservation.

## Ethics statement

The studies involving human participants were reviewed and approved by the Research Ethics Committee of the Tokyo Metropolitan Institute for Geriatrics and Gerontology. The patients/participants provided their written informed consent to participate in this study.

## Author contributions

TO designed the study, analyzed the data, and wrote the manuscript. HS, YO, NK, TA, MY, SO, TI, YF, SA, and IRIDE Cohort Study Investigators collected the data and revised the manuscript. KT supervised the project. All authors critically revised the manuscript for important intellectual content and approved the version to be published.
