# Supplementary material for: Age- and sex-specific associations between sarcopenia severity and poor cognitive function among community-dwelling older adults in Japan: The IRIDE Cohort Study
Source: Front Public Health. 2023 Apr 4;11:1148404. doi: 10.3389/fpubh.2023.1148404 (PMC10110951; doi:10.3389/fpubh.2023.1148404)
Supplement: Supplementary file 1 [file Table_1.DOCX]

Supplemental list. List of the IRIDE Cohort Study investigators.

# IRIDE cohort study

Kenji Toba, Shuichi Awata, Yoshinori Fujiwara, Tatsuro Ishizaki, Hiroyuki Sasai, Shuichi P. Obuchi, Takumi Abe, Mari Yamashita

# Otassha Study

Otassha study 2011: Shuichi P. Obuchi*, Hisashi Kawai^†^, Hirohiko Hirano^†^, Hunkyung

Kim^†^, Yoshinori Fujiwara^†^, Kazushige Ihara^†^, Manami Ejiri^†^, Yuki Ohara, Keiko

Motokawa, Maki Shirobe, Masanori Iwasaki, Hiroyuki Suzuki, Ryota Sakurai, Masahiro

Hashizume, Mitsugu Hachisu, Yutaka Watanabe, Hunkyung Kim, Motonaga Kojima,

Takeshi Kera, Akiko Miki, Junta Takahashi, Kumiko Ito

Otasha study 2017&2019: Hiroyuki Sasai*, Hunkyung Kim^‡^, Narumi Kojima^†^, Yosuke Osuka^†^, Takahisa Ohta

# Takashimadara Study

Shuichi Awata*, Hirohiko Hirano, Tsuyoshi Okamura, Masanori Iwasaki, Yuki Ohara,

Hiroki Inagaki, Chiaki Ura, Ayako Edahiro, Naoko Sakuma, Mika Sugiyama, Fumiko

Miyamae, Shuji Tsuda, Narumi Kojima, Yosuke Osuka, Keiko Motokawa, Hiroyuki

Suzuki, Hidenori Amano, Satoshi Seino, Ryota Sakurai, Yukie Masui, Hisashi Kawai,

Shoji Shinkai, Yutaka Watanabe, Akihiko Kitamura, Chiho Shimada, Hunkyung Kim, Madoka Ogawa, Yu Taniguchi

# SONIC study

Tatsuro Ishizaki*, Yukie Masui^‡^, Yasuyuki Gondo^‡^, Kazunori Ikebe^‡^, Kei Kamide^‡^, Yasumichi Arai^‡^, Yuri Miura, Hiroki Inagaki, Madoka Ogawa, Yuko Yoshida, Saori Yasumoto, Ayaka Kasuga, Kodai Hatta, Toshihito Takahashi, Masahiro Kitamura,

Shinya Murakami, Mai Kabayama, Kayo Godai, Hiroshi Akasaka, Hiromi Rakugi, Yoshiko Ishioka, Takeshi Nakagawa, Hiroyuki Muto

# Hatoyama Study

Yoshinori Fujiwara*, Akihiko Kitamura^‡^, Shoji Shinkai^‡^, Mariko Nishi, Hiroshi

Murayama, Yuri Yokoyama, Satoshi Seino, Yu Nofuji, Hidenori Amano, Miki Narita, Takumi Abe

# Kusatsu Longitudinal Study on Aging

Yoshinori Fujiwara*, Akihiko Kitamura^‡^, Shoji Shinkai^‡^, Yu Nofuji, Takumi Abe,

Hidenori Amano, Toshiki Hata, Hirohiko Hirano, Ai Iizuka, Tomoko Ikeuchi, Hiroshi

Murayama, Miki Narita, Mariko Nishi, Yuki Ohara, Ryota Sakurai, Satoshi Seino, Hiroyuki Suzuki, Yu Taniguchi, Mari Yamashita, Yuri Yokoyama

* Principal investigator

1. Core member
2. Co-principal investigator
