# Supplementary material for: Age- and sex-specific associations between sarcopenia severity and poor cognitive function among community-dwelling older adults in Japan: The IRIDE Cohort Study
Source: Front Public Health. 2023 Apr 4;11:1148404. doi: 10.3389/fpubh.2023.1148404 (PMC10110951; doi:10.3389/fpubh.2023.1148404)
Supplement: Supplementary file 2 [file Table_2.DOCX]

| Supplemental Table. Characteristics of the cohort according to cognitive status of different sexes. | | | | | |
| --- | --- | --- | --- | --- | --- |
|  |  | Men  (n=2220) | | Women  (n=4206) | |
|  |  | Cognitive status | | Cognitive status | |
|  | Overall  (n=6426) | Poor cognition  (n=127, 5.7%) | Normal cognition  (n=2093, 94.3%) | Poor cognition  (n=143, 3.4%) | Normal cognition  (n=4063, 96.6%) |
| Age, years | 74 [9] | 79 [9] | 74 [10] | 79 [7] | 73 [9] |
| 65–74 | 3438 (53.5) | 33 (26.0) | 1124 (53.7) | 31 (21.7) | 2250 (55.4) |
| ≥ 75 | 2988 (46.5) | 94 (74.0) | 969 (46.3) | 130 (90.9) | 1795 (44.2) |
| MMSE, score | 29 [3] | 22 [3] | 29 [2] | 22 [3] | 29 [2] |
| Sarcopenia status |  |  |  |  |  |
| Non-sarcopenia | 5311 (82.6) | 85 (66.9) | 1745 (83.4) | 80 (55.9) | 3401 (83.7) |
| Sarcopenia | 868 (13.5) | 34 (26.8) | 275 (13.1) | 41 (28.7) | 518 (12.8) |
| Severe sarcopenia | 258 (4.0) | 19 (15.0) | 73 (3.5) | 40 (28.0) | 126 (3.1) |
| Past Medical History |  |  |  |  |  |
| Hypertension | 2849 (44.3) | 65 (51.2) | 1009 (48.2) | 73 (51.1) | 1702 (41.9) |
| Diabetes | 811 (12.6) | 28 (22.1) | 376 (18.0) | 24 (16.8) | 383 (9.4) |
| Dyslipidemia | 2257 (35.1) | 22 (17.3) | 588 (28.1) | 49 (34.3) | 1598 (39.3) |
| Stroke | 377 (5.9) | 15 (11.8) | 178 (8.5) | 12 (8.4) | 172 (4.2) |
| Smoking |  |  |  |  |  |
| Current | 624 (9.7) | 15 (11.8) | 364 (17.4) | 7 (4.9) | 238 (5.9) |
| Past | 1623 (25.3) | 69 (54.3) | 1113 (53.2) | 20 (14.0) | 421 (10.4) |
| Never | 4065 (63.3) | 39 (30.7) | 578 (27.6) | 120 (83.9) | 3328 (81.9) |
| Alcohol Consumption |  |  |  |  |  |
| Current | 2844 (44.3) | 65 (51.2) | 1332 (63.6) | 40 (28.0) | 1407 (34.6) |
| Past | 518 (8.1) | 26 (20.5) | 220 (10.5) | 16 (11.2) | 256 (6.3) |
| Never | 2881 (44.8) | 24 (18.9) | 464 (22.2) | 91 (63.6) | 2302 (56.7) |
| Educational attainment, years | 12 [4] | 10 [4] | 12 [5] | 10 [3] | 12 [3] |
| GDS-15, score | 2 [3] | 3 [5] | 2 [3] | 4 [4] | 2 [3] |
| Depressive symptoms, n | 1244 (19.4) | 43 (33.9) | 396 (18.9) | 55 (38.5) | 750 (18.5) |
| Frequency of going outdoors (days per week) |  |  |  |  |  |
| < 1 | 39 (0.6) | 4 (3.2) | 14 (0.7) | 4 (2.8) | 17 (0.4) |
| 1–2 | 192 (3.0) | 7 (5.5) | 63 (3.0) | 13 (9.1) | 109 (2.7) |
| 3–6 | 1269 (19.7) | 24 (18.9) | 390 (18.6) | 41 (28.7) | 814 (20.0) |
| 7 | 4902 (76.3) | 90 (70.9) | 1620 (77.4) | 99 (69.2) | 3093 (76.1) |
| Exercise habits (days per week) |  |  |  |  |  |
| < 1 | 888 (13.8) | 16 (12.6) | 284 (13.6) | 20 (14.0) | 568 (14.0) |
| 1–4 | 5068 (78.9) | 93 (73.2) | 1589 (75.9) | 118 (82.5) | 3268 (80.4) |
| > 4 | 86 (1.3) | 0 (-) | 52 (2.5) | 3 (2.1) | 31 (0.8) |
| Data are means (standard deviations) or median [interquartile range], or numbers (percentages).  MMSE; Mini-mental State Examination, GDS; Geriatric Depression Scale-15,  Depressive symptoms were defined as a GDS score ≥ 5. | | | | | |
